# Association between *PNPLA2* Gene Polymorphisms and the Risk of Diabetic Kidney Disease in a Chinese Han Population with Type 2 Diabetes

**DOI:** 10.1155/2020/5424701

**Published:** 2020-06-30

**Authors:** Hailing Zhao, Haojun Zhang, Yan Wang, Tingting Zhao, Meihua Yan, Xi Dong, Qian Wang, Jialin Li, Liang Ma, Ping Li

**Affiliations:** ^1^Beijing Key Lab for Immune-Mediated Inflammatory Diseases, Institute of Clinical Medical Science, China-Japan Friendship Hospital, Beijing, China; ^2^Beijing Key Laboratory of Diabetes Research and Care, Center for Endocrine Metabolism and Immune Diseases, Luhe Hospital, Capital Medical University, Beijing, China; ^3^Clinical Laboratory, China-Japan Friendship Hospital, Beijing, China

## Abstract

Diabetic kidney disease (DKD) is one of the most common complications of diabetes and the leading cause of end-stage renal disease. Here, we investigated the association of *PNPLA2* gene variations with DKD susceptibility in a Chinese Han population. A total of 818 participants with type 2 diabetes were recruited in the case-control study, including 379 patients diagnosed with DKD. We observed that 2 tagSNPs, *PNPLA2* rs28633403 (A>G) and rs1138714 (A>G), were associated with DKD (rs28633403: genotype, *P* = 0.017; allele, *P* = 0.015; rs1138714: genotype, *P* = 0.029; allele, *P* = 0.018). *PNPLA2* rs1138693 (T>C), a missense SNP, showed no association with DKD (genotype, *P* = 0.966; allele, *P* = 0.845). Genetic model analysis revealed that minor allele G of *PNPLA2* rs28633403 was a protective factor of DKD in a dominant model adjusted by confounders (AG+GG vs. AA: adjusted odds ratio (aOR), 0.619; 95% CI 0.447-0.857; *P* = 0.004) and in an additive model (AG vs. AA: aOR, 0.633; 95% CI 0.447-0.895; *P* = 0.010; GG vs. AA: aOR, 0.588; 95% CI 0.385-0.897; *P* = 0.014). Minor allele G of *PNPLA2* rs1138714 was associated with a higher risk of DKD in a dominant model adjusted by confounders (AG+GG vs. AA: adjusted odds ratio (aOR), 1.531; 95% CI 1.134-2.067; *P* = 0.005) and in an additive model (AG vs. AA: aOR, 1.529; 95% CI 1.118-2.091; *P* = 0.008). The combined effect of *PNPLA2* rs28633403 AA+rs1138714 AG or GG genotype showed an association with DKD, adjusted by confounders (aOR, 2.194; 95% CI 1.378-3.492; *P* = 0.001), which was considered statistically significant with a markedly increased risk of DKD after a Holm-Bonferroni correction for multiple tests (*P* < 0.00125). Our results suggest that *PNPLA2* rs28633403 and rs1138714 are significantly associated with the risk of DKD in a Chinese Han population with type 2 diabetes.

## 1. Introduction

Diabetic kidney disease (DKD) is one of the most common microvascular complications of diabetes mellitus (DM) characterized by an increased urinary albumin excretion rate and declined renal function [1]. Approximately 40% of DM patients develop DKD; therefore, DKD is the leading cause of end-stage renal disease (ESRD) and renal failure [2]. According to the International Diabetes Federation (IDF) survey in 2017, there are 451 million (age 18-99 years) people worldwide suffering from diabetes mellitus, and the number is expected to increase to 693 million by 2045 [3]. The pathogenesis of DKD is relatively complex, and there is growing evidence for genetic factors contributing to DKD susceptibility. Many studies have provided compelling data that DKD has genetic tendency, and familial aggregation additionally indicates that genetic factors play an important role in the etiology of the disease [4, 5]. Genetic research may help to reveal the pathobiology of DKD and uncover potential targets for its treatment.

Recent studies have shown accumulation of fatty acids (FAs) and triglycerides (TGs) in the kidneys of patients with DKD, in a diabetic animal model, and in tubular cells exposed to high-glucose (HG) conditions, which thereby lead to kidney dysfunction [6–8]. Adipose triglyceride lipase (ATGL), encoded by the *PNPLA2* gene, is an important component of the lipolytic process and the rate-limiting enzyme for the initiation of TG catabolism [9, 10]. It is reported that ATGL deficiency can impair the renal fatty acid metabolism, which may lead to renal lipid accumulation, proteinuria, and glomerular filtration barrier dysfunction [11, 12]. The relationship between ATGL and kidney injuries in diabetic patients remains unclear.

*PNPLA2* is a susceptibility gene of nonalcoholic fatty liver disease (NAFLD) in an obese population [13]. Rs28633403 (A>G) and rs1138714 (A>G) are tagSNPs in *PNPLA2* gene with the frequency of minor allele greater than 5%, and rs1138693 (T>C) is a missence polymorphism (L481P) in coding sequence. Genetic studies in obese individuals have shown that rs1138714 was associated with fat mass percentage and volume of subcutaneous adipose tissue, and rs28633403 was related to fat mass percentage and subcutaneous adipose tissue. For SNP rs1138693, it is a risk factor for susceptibility to increased levels of Aminotransferase (AST) enzyme [13]. However, the association of the SNPs with DKD is still unclear. In this study, we selected these 3 SNPs in *PNPLA2* gene to explore their association with DKD in type 2 diabetic patients.

To the best of our knowledge, this is the first case-control association study to explore the role of *PNPLA2* in the pathogenesis of DKD. In this study, we aimed to evaluate the contribution of *PNPLA2* gene polymorphisms to the progression of DKD in patients with type 2 diabetes in a Chinese Han population.

## 2. Materials and Methods

### 2.1. Clinical Samples

This was a case-control study of 818 patients with type 2 diabetes (age 36-85 years), diagnosed according to the 2012 American Diabetes Association diagnostic criteria. Individuals with a history of DKD were defined as the case group (*n* = 379). All patients with DKD were based on the National Kidney Foundation Kidney Disease Outcomes Quality Initiative (NKF/KDOQI) guidelines. The remaining 439 participants were defined as the controls (*n* = 439), diagnosed with type 2 diabetes for at least 7 years and no history of DKD. This study was approved by the institutional ethics committee of the China-Japan Friendship Hospital (Beijing, China), and the number of the ethical commission statement was 2016-59. Written informed consent was obtained from all individuals.

### 2.2. DNA Isolation and Genotyping

Genomic DNA was extracted from peripheral blood using a QIAamp DNA Blood Mini Kit (Qiagen, Hilden, Germany), and DNA quality was measured using a Nanodrop 1000 spectrophotometer (ThermoScientific, Waltham, MA, USA). Genotyping was confirmed by polymerase chain reaction (PCR) using the TaqMan SNP Genotyping Assay (Applied Biosystems, Waltham, MA, USA) and the ABI PRISM 7500 Sequence Detection System (Applied Biosystems). The amplification conditions were determined based on our previous research [14].

Genotyping was verified by randomly selected PCR products for DNA sequencing analysis by TsingKe Biological Technology (Beijing, China). The primers used for the PCR were the following: rs28633403, 5′-CCAGAAGAATGCGAACGG-3′ (forward) and 5′-CCCTGATTACCC AAACTCC-3′ (reverse); rs1138714, 5′-AGAGGGGTCTTTGCCGTGG-3′ (forward) and 5′-GCAAGTAAGCAGGCGGTCAC-3′ (reverse); and rs1138693, 5′-GCTGCTGCTCGGCCTC TTCT-3′ (forward) and 5′-AGGCGTCTCAGGCAGGGTTC-3′ (reverse).

### 2.3. Statistical Analyses

The baseline characteristics were non-Gaussian distributed, including age, gender, body mass index (BMI), blood pressure, duration of diabetes, hemoglobin levels (A1C), total cholesterol (TC), high-density lipoprotein cholesterol (HDL-C), low-density lipoprotein cholesterol (LDL-C), triglyceride (TG), and homocysteine (Hcy). A Wilcoxon signed rank test was used to analyze clinical characteristics of the DKD and control groups, and the data were presented as a median (interquartile range). The Hardy-Weinberg equilibrium of SNPs was analyzed using the Chi-squared test. The genotype and allelic frequencies of SNPs were also assessed using the Chi-squared test.

In the additive, recessive, or dominant models, we used binary logistic regression to analyze the association between 3 SNPs and DKD by calculating odds ratios (ORs) and 95% confidence intervals (CIs). The logistic regression analysis data were adjusted for confounders showing significant differences in the T2DM and DKD groups, including age, BMI, blood pressure, A1C, Hcy, TC, and TG. Logistic regression analysis was also used for the combined effect of both *PNPLA2* rs28633403 (A>G) and rs1138714 (A>G) polymorphisms on DKD. We considered the PNPLA2 rs28633403 as an example to define these genetic models, where G is the minor allele. For the dominant model, GG and AG were coded as 1, and AA was coded 0. For the recessive model, GG was coded as 1, and AG and AA were coded as 0. For the additive model, GG, AG, and AA were coded as 2, 1, and 0, respectively. For SNP rs1138714 (A>G), G is the minor allele. The statistical analysis was carried out by following our previous research [14]. For all statistical tests of this study, statistical analysis was performed with SPSS software (version 20.0), and two-tailed *P* values less than 0.05 were considered statistically significant.

Correction for multiple testing was carried out by the Holm-Bonferroni correction. The *P* values associated with genotype analysis were ordered from lowest to highest as *P*1 ⋯ *Pm*, and the associated hypotheses were to be *H*1 ⋯ *Hm*. If *α* is the significance level, let *κ* be the minimal index such that *P* > *α*/*m* + 1 − *κ*. The null hypotheses *H*1 ⋯ *Hκ* − 1 were rejected, and *Hκ* ⋯ *Hm* were not rejected. In this study, *α* was equal to 0.05, and association analysis was performed for 40 times, meaning that *m* is equal to 40.

## 3. Results

### 3.1. Analyses of Clinical Samples

A total of 818 type 2 diabetes mellitus (T2DM) participants were recruited, including 379 patients with a history of DKD and 439 patients without a history of kidney disease. The clinical characteristics of the participants are listed in Table 1. There were no significant differences in sex, duration of diabetes, smoking, HDL-C, and LDL-C between the two groups. However, there were significant differences in age, BMI, blood pressure, A1C, Hcy, TC, and TG parameters between the DKD and T2DM groups.

### 3.2. Genotype and Allele Distributions of *PNPLA2* Polymorphisms

The genotype and allele frequencies of the *PNPLA2* rs28633403 (A>G), rs1138714 (A>G), and rs1138693 (T>C) polymorphisms in T2DM and DKD groups were obtained and presented in Table 2. The distribution of allele frequencies of 3 SNPs was in accordance with the Hardy-Weinberg equilibrium (*P* > 0.05) and the minor allele frequencies were all greater than 5% in the Chinese Han population, suggesting the suitability of this population for genetic analysis.

The genotype and allele frequencies of *PNPLA2* rs28633403 (A>G) and rs1138714 (A>G) were significantly different in the DKD and T2DM groups (rs28633403: genotype, *P* = 0.017; allele, *P* = 0.015; rs1138714: genotype, *P* = 0.029; allele, *P* = 0.018). For *PNPLA2* rs1138693 (T>C) polymorphism, no significant difference in the genotype and allele frequencies was observed in the two groups (genotype, *P* = 0.966; allele, *P* = 0.845) (Table 2).

### 3.3. Association of *PNPLA2* Polymorphisms with DKD

To define the contribution of *PNPLA2* polymorphisms to the risk of DKD, genetic model analyses using binary logistic regression assessment after adjustment with confounders were performed and summarized in Table 3. Compared to the common alleles, the minor alleles of 3 SNPs were assumed to be the risk factors for DKD.

We determined that *PNPLA2* rs28633403 was significantly associated with decreased risk of DKD in the additive and dominant models, respectively (additive models: GG vs. AA, adjusted OR = 0.588, 95% CI 0.385-0.897, *P* = 0.014; AG vs. AA, adjusted OR = 0.633, 95% CI 0.447-0.895, *P* = 0.010; dominant models: adjusted OR = 0.619, 95% CI 0.447-0.857, *P* = 0.857, *P* = 0.004). For SNP *PNPLA2* rs1138714, the AG genotype in the additive model had 1.529-fold increased risk of DKD (adjusted OR = 1.529, 95% CI 1.118-2.091, *P* = 0.008), and the AG+GG genotype in the dominant model showed 1.531-fold increased risk of DKD (adjusted OR = 1.531, 95% CI 1.134-2.067, *P* = 0.005).

### 3.4. Combined Effect of *PNPLA2* rs28633403 (A>G) and rs1138714 (A>G) Polymorphisms on DKD

Since no significant association was found between *PNPLA2* rs1138693 and the risk of DKD, only *PNPLA2* rs28633403 (A>G) and rs1138714 (A>G) were used to perform the combination risk analysis. The combined effect of both SNPs on the risk of DKD was analyzed by binary logistic regression analysis (Table 4). The patients with both protective genotypes *PNPLA2* rs28633403 GG and rs1138714 AA were used as reference. We found that the patients with rs28633403 AA and rs1138714 (AG+GG) genotype showed a higher risk of DKD (adjusted OR, 2.194; 95% CI 1.378-3.492; *P* = 0.001). After the Holm-Bonferroni correction, significant differences remained (*P* < 0.00125). The combined effects of other genotypes of rs28633403 and rs1138714 were also examined; the results showed that compared with the reference, other genotypes were not a high risk factor for DKD (Table 4).

## 4. Discussion

Lipotoxicity and ectopic lipid accumulation in the kidney play a role in the pathogenesis of DKD [15]. ATGL, encoded by the *PNPLA2* gene, is the rate-limiting enzyme for the initiation of TG catabolism and is essential for the lipid homeostasis [16]. In the present study, 818 participants (439 T2DM patients and 379 DKD patients) were enrolled to investigate the susceptibility of *PNPLA2* polymorphisms with the risk of DKD in a Chinese Han population. We found significant difference in genotype frequencies of *PNPLA2* rs28633403 (A>G) and rs1138714 (A>G) between T2DM and DKD patients. A higher frequency of rs28633403 A allele and rs1138714 G allele was shown in the DKD patients than in the T2DM patients. In the genetic model, when the allele G of *PNPLA2* rs28633403 is the dominant, T2DM patients with GG or AG genotypes showed lower risk of DKD in the additive and dominant models. For *PNPLA2* rs1138714, there is a higher risk of DKD for T2DM patients with AG genotypes in the additive model and with AG or GG in the dominant model. The patients with both rs28633403 AA and rs1138714 AG or GG genotype showed a higher risk of DKD. After Holm-Bonferroni correction, patients with rs28633403 AA and rs1138714 (AG+GG) genotype showed a higher risk of DKD. The results suggest that *PNPLA2* rs28633403 and rs1138714 might play an important role in the risk of DKD in the Chinese Han population.

Dyslipidemia is a reversible risk factor for the progression of kidney disease and cardiovascular mortality in patients with type 2 diabetes [17]. Sustained hyperglycemia in diabetes accelerates FA synthesis and TG accumulation. Elevated serum levels of TGs, FFAs, and modified cholesterol lead to ectopic lipid accumulation in nonadipose tissues such as the kidney, which can further cause renal dysfunction, fibrosis, and glomerulosclerosis, finally resulting in DKD [18–20]. Despite of reported evidence of lipid accumulation and lipotoxicity in kidneys in human and animal DKD models, the underlying molecular mechanism remains unclear. Epidemiological studies have shown the existence of a genetic susceptibility to the development of DKD [21]. Interestingly, previous studies have revealed the role of lipid metabolism-related genes in the lipid deposition, resulting in the decline of the glomerular filtration rate in DKD [22, 23]. For example, variants in the acetyl-coenzyme A carboxylase beta (ACACB) gene have been likely involved in the development of DKD. An intron SNP rs2268388 in ACACB showed a significant association with type 2 diabetic kidney disease in Japanese individuals, and the strongest associations have been confirmed by case-control studies in Asian, including Chinese and Caucasian, populations [24–26]. Therefore, finding susceptibility genes for DKD might be an efficient strategy to identify candidates for DKD therapy. In this article, we demonstrated *PNPLA2* rs28633403 and rs1138714 to be significantly associated with DKD in the Chinese Han population with type 2 diabetes. Further functional studies are necessary to determine whether the risk alleles affect physiological activity of ATGL, causing renal lipid deposition.

The human *PNPLA2* gene, located on chromosome 11p15.5, was first discovered in 2004 by three independent laboratories [10, 27, 28]. *PNPLA2* includes 10 exons encoding a 504 amino acid protein, named ATGL [29]. ATGL is expressed in almost all tissues but mainly in adipose tissue. ATGL, as the initial rate-limiting step for TG hydrolysis, plays a key role in maintaining the dynamic balance of lipid metabolism [16]. Normally, FAs are recruited to form TGs stored in lipid droplets. When energy demand increases, ATGL plays an important role in the initial step of catalyzing the hydrolysis of TG to diacylglycerol (DG) and FA. Subsequently, DG is hydrolyzed by hormone-sensitive lipase (HSL) into monoacylglycerol (MG) and FA, while monoglyceride lipase (MGL) cracks MG into glycerol and FA. Finally, the released FAs will serve as the substrates for energy production. Abnormal lipolysis leads to the increased levels of circulating FAs, which cause lipotoxicity, including insulin resistance, type 2 diabetes, fatty liver, and inflammation [30]. Haemmerle et al. reported for the first time that TG content in myocytes of ATGL-deficient (Atgl (-/-)) mice, compared with Atgl (+/+) mice, was increased by more than 20-fold [9]. Since then, many studies have been conducted to explore the relationship between ATGL and cardiac dysfunction [31, 32]. For the kidney, accumulation of TG was increased by more than 10-fold in Atgl (-/-) mice. ATGL deficiency induced renal lipid accumulation, proteinuria, and glomerular filtration barrier dysfunction. In the podocytes and proximal tubular cells, ATGL deletion impaired intracellular fatty acid metabolism and increased reactive oxygen species levels and apoptosis, aggravating thereby cell dysfunction [11, 12]. A recent study demonstrated for the first time a significant decrease of ATGL activity in the kidney of diabetic mice, similarly to the decreased levels of ATGL in human kidney-2 (HK-2) cells exposed to HG compared with the cells cultured under low-glucose conditions [33]. These studies suggest that finding the regulatory mechanism of ATGL activity might provide a new strategy for the treatment of DKD.

Our study found that the two *PNPLA2* polymorphisms rs28633403 and rs1138714 showed significant association with DKD, and both SNPs are located in noncoding regions of *PNPLA2*. Previous studies have shown that most disease-associated SNPs are located in the noncoding region, suggesting that they may play a regulatory role in phenotypes [34]. Among them, the SNPs that change the binding affinity of transcription factors or miRNA and affect gene expression constitute an important class of regulatory SNPs [35]. In our study, SNP rs28633403, located at 5.6 kb upstream of *PNPLA2* gene on chromosome 11, was found to be a tagSNP of *PNPLA2* gene [13]. The effects of transcription factor binding and methylation levels might be involved in the underlying mechanism by which *PNPLA2* rs28633403 leads to DKD susceptibility. Rs1138714 was located in the 3′UTR region of *PNPLA2* gene, and variation of alleles might affect posttranscriptional regulation of *PNPLA2* by regulating miRNA binding and then changing the expression level of ATGL, which may be the cause of DKD progression. In addition, these two SNPs might not be the causal variation but are in a strong linkage with the causal variation. Of course, more research is needed in the future to determine the true mechanism by which *PNPLA2* polymorphisms lead to DKD susceptibility. SNP rs1138693 is a missense variant located in the coding region of *PNPLA2* gene, and the allele C to T changes the amino acid from leucine to proline, which might affect the activity of ATGL. Although our study found no correlation between rs1138693 and DKD susceptibility, the effect of missense variation on ATGL activity is worth further exploration.

Nevertheless, there are some potential limitations, which should be considered for this study. First, more people with type 2 diabetes should be recruited to form a larger study cohort, which will improve the statistical power. Second, although the two tagSNPs in *PNPLA2* gene were identified as susceptibility variants of DKD in patients with type 2 diabetes in a Chinese Han population, it is important to screen more sites in *PNPLA2* gene to clarify the interaction between SNPs and DKD. Third, since this is the first study to explore the association between *PNPLA2* gene and DKD, our results need to be replicated in other independent cohorts in the future. Finally, the potential underlying mechanism, by which rs28633403 and rs1138714 lead to susceptibility of DKD, should be investigated in future studies for searching genotype-phenotype correlations between *PNPLA2* and DKD.

In conclusion, our study suggests that A allele of *PNPLA2* rs28633403 and G allele of rs1138714 are significantly associated with risk of DKD in patients with type 2 diabetes in a Chinese Han population. To our knowledge, this is the first study to identify the correlation between *PNPLA2* and DKD; therefore, large, well-designed replication studies in China and other populations are needed to verify our findings. In future investigations, it is important to pay more attention on the underlying mechanisms of this association.

## Figures and Tables

**Table 1:** Demographics and clinical characteristics of T2DM patients with and without kidney diseases.

Variables	DM (*n* = 439)^a^	DKD (*n* = 379)^a^	*P*
Age (y)	61.0 (54.0, 68.0)	63.0 (54.0, 72.0)	0.001
Sex, male (%)	58.77 (258/439)	62.80 (238/379)	0.240
BMI (kg/m^2^)	25.34 (23.20, 27.68)	25.80 (24.0, 28.23)	0.007
Duration of diabetes (y)	13.0 (10.0, 18.0)	14.0 (9.0, 20.0)	0.242
History of hypertension (%)	49.66 (218/439)	78.89 (299/379)	<0.001
Currently smoking (%)	27.79 (122/439)	33.51 (127/379)	0.076
SBP (mmHg)	126.0 (120.0, 140.0)	138.0 (125.00, 150.0)	<0.001
DBP (mmHg)	80.0 (70.0, 80.00)	80.0 (74.0, 84.0)	0.032
A1C (%)	7.90 (6.70, 9.30)	7.50 (6.40, 9.20)	0.025
Hcy (*μ*mol/L)	11.46 (9.50, 13.42)	13.44 (10.70, 16.94)	<0.001
TC (mmol/L)	4.14 (3.50, 4.86)	4.25 (3.49, 5.06)	0.031
HDL-C (mmol/L)	1.01 (0.84, 1.22)	0.96 (0.78, 1.18)	0.185
LDL-C (mmol/L)	2.38 (1.93, 2.97)	2.39 (1.87, 3.00)	0.690
TG (mmol/L)	1.43 (1.00, 2.19)	1.72 (1.22, 2.57)	<0.001

Abbreviations: BMI: body mass index; SBP: systolic blood pressure; DBP: diastolic blood pressure; A1C: hemoglobin A1C; Hcy: homocysteine; TC: total cholesterol; HDL-C: high-density lipoprotein cholesterol; LDL-C: low-density lipoprotein cholesterol; TG: triglyceride. ^a^Data are shown as median (interquartile range) or %. *P* < 0.05 indicates statistical significance.

**Table 2:** Genotype and allele frequency of SNPs in *PNPLA2* between DM controls (*n* = 439) and DKD patients (*n* = 379).

	Genotype frequencies						Allele frequencies		
*PNPLA2* rs28633403	AA	AG	GG	HWE *P* value	*P*		A	G	*P*
DM	117 (26.7%)	224 (51.0%)	98 (22.3%)	0.639	0.017^∗^		458 (52.2%)	420 (47.8%)	0.015^∗^
DKD	136 (35.9%)	169 (44.6%)	74 (19.5%)	0.103			441 (58.2%)	317 (41.8%)	
*PNPLA2* rs1138714	AA	AG	GG	HWE *P* value	*P*		A	G	*P*
DM	225 (51.3%)	180 (41.0%)	34 (7.7%)	0.809	0.029^∗^		630 (71.8%)	248 (28.2%)	0.018^∗^
DKD	159 (42.0%)	185 (48.8%)	35 (9.2%)	0.069			503 (66.4%)	255 (33.6%)	
*PNPLA2* rs1138693	TT	TC	CC	HWE *P* value	*P*		T	C	*P*
DM	178 (40.5%)	204 (46.5%)	57 (13.0%)	0.903	0.966		560 (63.8%)	318 (36.2%)	0.845
DKD	157 (41.4%)	173 (45.6%)	49 (12.9%)	0.901			487 (64.2%)	271 (35.8%)	

^∗^ *P* < 0.05 indicates statistical significance. HWE: Hardy-Weinberg equilibrium.

**Table 3:** Genetic model analyses of the association between *PNPLA2* polymorphisms and DKD with adjustment for confounders.

	Genetic models	Genotype	DM	DKD	Without adjustment		With adjustment^¶^	
					OR (95% CI)	*P*	OR (95% CI)	*P*
*PNPLA2* rs28633403	Additive	AA	117 (26.7%)	136 (35.9%)	1^#^	**—**	1^#^	**—**
		AG	224 (51.0%)	169 (44.6%)	0.649 (0.472-0.892)	0.008^∗^	0.633 (0.447-0.895)	0.010^∗^
		GG	98 (22.3%)	74 (19.5%)	0.650 (0.440-0.960)	0.030^∗^	0.588 (0.385-0.897)	0.014^∗^
	Dominant	AA	117 (26.7%)	136 (35.9%)	1^#^	**—**	1^#^	—
		AG+GG	322 (73.3%)	243 (64.1%)	0.649 (0.482-0.875)	0.004^∗^	0.619 (0.447-0.857)	0.004^∗^
	Recessive	AA+AG	341 (77.7%)	305 (80.5%)	1^#^	—	1^#^	—
		GG	98 (22.3%)	74 (19.5%)	0.844 (0.601-1.185)	0.328	0.778 (0.540-1.121)	0.177

*PNPLA2* rs1138714	Additive	AA	225 (51.3%)	159 (42.0%)	1^#^	—	1^#^	—
		AG	180 (41.0%)	185 (48.8%)	1.454 (1.090-1.941)	0.011^∗^	1.529 (1.118-2.091)	0.008^∗^
		GG	34 (7.7%)	35 (9.2%)	1.457 (0.871-2.435)	0.151	1.545 (0.888-2.688)	0.124
	Dominant	AA	225 (51.3%)	159 (42.0%)	1^#^	—	1^#^	
		AG+GG	214 (48.7%)	220 (58.0%)	1.455 (1.103-1.919)	0.008^∗^	1.531 (1.134-2.067)	0.005^∗^
	Recessive	AA+AG	405 (92.3%)	344 (90.8%)	1^#^	—	1^#^	—
		GG	34 (7.7%)	35 (9.2%)	1.212 (0.740-1.985)	0.445	1.257 (0.739-2.138)	0.399

*PNPLA2* rs1138693	Additive	TT	178 (40.5%)	157 (41.4%)	1^#^		1^#^	
		TC	204 (46.5%)	173 (45.6%)	0.961 (0.716-1.292)	0.794	0.915 (0.663-1.263)	0.590
		CC	57 (13.0%)	49 (12.9%)	0.975 (0.629-1.510)	0.908	0.918 (0.573-1.469)	0.720
	Dominant	TT	178 (40.5%)	157 (41.4%)	1^#^	—	1^#^	—
		TC+CC	261 (59.5%)	222 (58.6%)	0.964 (0.729-1.275)	0.799	0.916 (0.675-1.242)	0.572
	Recessive	TT+TC	382 (87.0%)	330 (87.1%)	1^#^	—	1^#^	—
		CC	57 (13.0%)	49 (12.9%)	0.995 (0.661-1.498)	0.981	0.962 (0.621-1.491)	0.864

Abbreviations: ORs: odds ratios; CI: confidence interval. ^#^Reference category (odds ratio, 1.0); adjustment for age, BMI, SBP, DBP, A1C, Hcy, TC, and TG; ^∗^*P* value < 0.05 indicates statistical significance.

**Table 4:** The combined effect of *PNPLA2* rs28633403 and rs1138714 polymorphisms on DKD.

Genotypes		DM	DKD	Without adjustment		With adjustment^¶^	
*PNPLA2* rs28633403	*PNPLA2* rs1138714	439	379	OR (95% CI)	*P*	OR (95% CI)	*P*
GG	AA	91	66	1^#^	—	1^#^	—
AG	AA	98	69	0.971 (0.624-1.510)	0.895	1.049 (0.651-1.690)	0.844
AA	AA	36	24	0.919 (0.501-1.685)	0.785	0.836 (0.432-1.618)	0.595
GG	AG+GG	7	8	1.576 (0.544-4.561)	0.402	1.270 (0.415-3.889)	0.676
AG	AG+GG	126	100	1.094 (0.725-1.651)	0.668	1.140 (0.730-1.780)	0.563
AA	AG+GG	81	112	1.906 (1.244-2.921)	0.003^∗^	2.194 (1.378-3.492)	0.001^∗^^,*Δ*^

Abbreviations: ORs: odds ratios; CI: confidence interval. ^#^Reference category (odds ratio, 1.0); adjustment for age, BMI, SBP, DBP, A1C, Hcy, TC, and TG; ^∗^*P* value < 0.05 indicates statistical significance. *Δ* indicates statistical significance by Holm-Bonferroni correction.

## Data Availability

The data used to support the findings of this study are available from the corresponding authors upon reasonable requests.
